# Retracted: Clinical Efficacy and Safety of Percutaneous Spinal Endoscopy versus Traditional Open Surgery for Lumbar Disc Herniation: Systematic Review and Meta-Analysis

**DOI:** 10.1155/2023/9879237

**Published:** 2023-02-02

**Authors:** Journal of Healthcare Engineering

*Journal of Healthcare Engineering* has retracted the article titled “Clinical Efficacy and Safety of Percutaneous Spinal Endoscopy versus Traditional Open Surgery for Lumbar Disc Herniation: Systematic Review and Meta-Analysis” [1] due to concerns that the peer review process has been compromised.

Following an investigation conducted by the Hindawi Research Integrity team [2], significant concerns were identified with the peer reviewers assigned to this article; the investigation has concluded that the peer review process was compromised. We therefore can no longer trust the peer review process, and the article is being retracted with the agreement of the Chief Editor.

The authors do not agree to the retraction.
